# Effect of different surgical positions on intraocular pressure: a cross-sectional study

**DOI:** 10.1186/s12886-022-02547-z

**Published:** 2022-07-26

**Authors:** Yuhong Sun, Juan Wang, Wei Wang, Guohui Fan, Sinan Wu, Fei Zhao, Yi Lu, Di Liu, Yan Li, Jin Hu, Lin Yang, Yu Bai, Tong Zhao, Ying Zhao

**Affiliations:** 1 Department of Anesthesiology, China-Japan Friendship Hospital, Beijing, 100029 People’s Republic of China; 2 Beijng Tongren Hospital, Beijing, 100730 People’s Republic of China; 3 Department of Pharmacy, Beijing Hospital, National Center of Gerontology,, Institute of Geriatric Medicine, Chinese Academy of Medical Sciences, Beijing, 100730 People’s Republic of China

**Keywords:** Intraocular pressure, Influencing factors, Lithotomy, Head-down position, Prone position, Lateral position, Supine position

## Abstract

**Background:**

Intraoperative intraocular pressure (IOP) elevation is a risk factor for postoperative blindness. Surgical position is associated with intraoperative IOP elevation. In China, there are few studies on the effect of various surgical positions on intraoperative IOP. This study was conducted to explore IOP change and its related factors in four common surgical positions in China.

**Methods:**

This was a cross-sectional observational study. A total of 325 surgical patients who had non-ocular surgery from January 2019 to December 2019 in the hospital, were enrolled in this study. During their surgeries for general anesthesia, these participants were placed in lithotomy position/lateral position/prone position/supine position according to their surgery requirement. IOP was measured by icareTA03 handheld portable tonometer at 9 different time points from admission to exiting the operation room. And general information, postural position, and surgery information were collected through a uniform questionnaire. Multivariate analysis was performed to explore the related factors of IOP change.

**Results:**

IOP of both eyes on lithotomy position, lateral position, and supine position showed statistical differences by ANOVA test at each time point (p < 0.05). IOP of both eyes in the prone position before exit from the operating room was significantly higher than IOP 10-min after anesthesia (*p* < 0.01). IOP under different postural angles showed statistical differences (F value = 4.85, *P* < 0.05), and the larger the head-down angle, the higher the IOP. IOP on the compressed side in the lateral position was higher than that on the non-compressed side (*p* < 0.01). In the multivariate linear regression analysis adjusted by other factors, postural position and baseline IOP were associated with IOP difference between before and after surgery (*p* < 0.01).

**Conclusion:**

IOP in the four surgical positions showed different change patterns with the surgical process and position change. Nurses should assist the surgeon to reduce the head-down angle without interfering with the surgical operation and strengthen the inspection of IOP on patients with long-time surgery, to avoid intraoperative rapid IOP changes.

## Background

Different surgical sites, types, and methods require different surgical positions. When patients maintain passively the position required for surgery under general anesthesia for a long time, they lose their protective response and are unable to adjust their body posture autonomously. This affects not only the central nervous system, cardiovascular system, and respiratory system, but also the normal regulation function of the eyes.

In recent years, cases of postoperative visual loss (POVL) with non-ophthalmic surgery have been reported frequently, especially after prone spine surgery [1–3]. POVL is a postoperative complication with an incidence of 0.015% to 0.2% of post-surgical complications and is gradually being taken seriously by medical practitioners [4]. Although the incidence is extremely low, the prognosis is extremely poor. The most common neuro-ophthalmologic causes of POVL are ischemic optic neuropathies (ION) [5, 6]. Multiple factors have been proposed as risk factors for perioperative ION, including the long duration in the prone position, excessive blood loss, excessive fluid replacement, use of vasoconstricting agents, and head positioning [7].

Many studies indicated that the surgeries, especially with prolonged prone positioning and/or hypotensive anesthesia, can induce IOP changes and ocular perfusion imbalance [8, 9]. These rapid fluctuations in IOP and perfusion, play a role in the pathogenesis of the visual field defects and associated ocular morbidity that frequently complicate otherwise uneventful surgeries [10]. And when IOP approaches or exceeds pathological IOP (24 mm Hg) levels, patients have risks of visual damage [11]. Therefore, careful documentation and intraoperative ocular protection have received a great deal of attention from operating room nurses.

The placement of surgical positions is a necessary specialty skill for operating room nurses. The clinical positions of flat, head-low-foot-high lithotomy, lateral and prone are common. The current studies on intraoperative IOP mostly focused on the prone position in spinal surgery and head-low-foot-high position in gynecological surgery [12], while there were few studies of IOP comparison among four surgical positions of lithotomy position, lateral position, prone position, and supine position. Therefore, in this study, IOP measurements were performed at different time points in these four clinical surgical positions to understand the IOP change pattern and related factors, that might provide evidence for nursing interventions.

## Methods

### Study participants

This study was a cross-sectional observational study, which was approved by the ethics committee in China-Japan friendship hospital in 2017(No.2017–92) and adhered to the tenets of the Declaration of Helsinki.

A convenience sampling method was used to enroll 325 patients undergoing surgery in the operating room from January 2019 to December 2019 who required placement in the supine position, head-down lithotomy position, lateral position, or prone position for general anesthesia.

### Inclusion criteria

1) age ≥ 18 years; 2) good cardiopulmonary function without metabolic diseases, serious circulatory diseases, respiratory diseases, and infectious diseases; 3) selection of patients with ASA ratings I to II according to the American Society of Anesthesiologists' physical condition grading (ASA) criteria; 4) stable intraoperative vital signs and no serious complications; 5) duration of a surgery over 1 h (excluding anesthesia time).

### Exclusion criteria

1) high power myopia (> -600D); 2) preoperative seated IOP higher than 21 mmHg; 3) ocular diseases or history of conjunctivitis, intra-bulbar infection, etc.; 4) goiter and hyperthyroidism; 5) used intraocular pressure drugs within 2 weeks, such as glaucoma eye deops and oral medication; 6) intraoperative mechanical ventilation with mean airway pressure higher than 25 cmH_2_O (1 cm H_2_O = 0.098 kPa); 7) patients with multiple systemic trauma, fracture, shock, or Injury Severity Score (ISS) > 18; 8) patients with systemic diseases, such as chronic obstructive bronchitis, emphysema, liver and kidney dysfunction, cardiac insufficiency, and autoimmune diseases; and 9) emergency rescue during operation.

### Procedures

In this study, all participants before the enrollment signed the written informed consent. All participants were treated with tracheal intubation general anesthesia and the anesthesia process was the same. After obtaining the intravenous access in the operating room, anesthetic drugs were given sequentially: midazolam 2–3 mg, propofol 2–2.5 mg/kg, cis-atracurium 0.2 mg/kg, and fentanyl 2-4ug/kg. After oral endotracheal intubation, remifentanil 0.1–0.2 ug/kg*min and propofol 4–6 mg/kg*h were added during operation, and CIS-Atracurium 0.05 ~ 0.1 mg/kg was added according to the operation time. PetCO2 was maintained in the range of 35–45 cmH_2_O by adjusting the minute-ventilation during operation. Remifentanil and cis-atracurium were discontinued 20 min before the surgery ended, and fentanyl 0.1 mg was administered. Propofol was discontinued 5 min before the end of surgery and the participant was taken to the anesthesia recovery room after surgery.

The IOP data collection form was designed to collect the information for this study, including 1) general information (name, gender, age, height, weight), 2) name of surgery and surgical position, 3) IOP at different points (T0: sitting IOP before admission, T1: after patient admission, T2: 10 min after giving general anesthesia in the supine position, T3: 10 min after changing position, T4: 1 h after the start of surgery, T5: 2 h after the start of surgery, T6: 4 h after the start of surgery, T7: 10 min before the end of the surgery, T8: before leaving the room), 4) heart rate, blood pressure, oxygen saturation, fluid volume, urine volume, blood loss, and vasoactive drugs from the start of surgery. IOP after admission was measured 10 min after entering the operating room; IOP after general anesthesia was measured 10 min after completion of tracheal intubation; IOP after position change was measured 10 min after the start of position adjustment; the start of surgery was recorded at the start of sterilization by the surgeon; the end of surgery was recorded at the completion of skin suturing by the surgeon; IOP before discharge was the IOP measured before the patient left the operating room after awake and extubate.

A total of six team members participating in this study were uniformly trained and tested for proficiency and standardization of the IOP meter, and those who were judged qualified by the ophthalmologist conducted the pilot study. The investigators used the icareTA03 handheld portable tonometer (Schiotz tonometer) to measure bilateral IOP at each point of surgery (One measurement procedure consisted of six measurements which were averaged as the measurement at this time point. On the other hand, the icareTA03 handheld portable tonometer (Schiotz tonometer) can not measure the IOP of patients in prone position, so IOP values in prone position from T3 and T7 were not measured in this study.) Vital signs, access volume, and vasoactive drugs were monitored and recorded at each time point hourly from the beginning of surgery. And intraoperative pneumoperitoneal pressure was recorded for laparoscopic surgery. The bed adjustment angle was accurately measured and recorded using a level meter. At the end of the operation, the patient was turned to the supine position, and the tracheal intubation was removed when the patient recovered consciousness, respiratory function, and good muscle strength.

The IOP meter was calibrated and quality-controlled by the designated person each morning. The measurement staff took the recorded IOP data and related information to the data fellows, who entered them into the statistical software in pairs and rejects any information that was missing from the intraoperative records or not recorded before leaving the operating room.

### Statistical analysis

SPSS19.0 statistical software was applied for processing. Mean ± standard deviation (x ± s) and range of IOP were analyzed in each position group. The change of IOP over time in each position group was analyzed by repeated-measures ANOVA; t-test or ANOVA was used for comparison between groups at a single time point; The proportion was calculated for the count data such as the number of patients in different groups and gender; the influencing factors of IOP change before and after surgery were analyzed by single-factor and multi-factor general linear regression. The difference was considered statistically significant at *P* < 0.05.

## Results

### General information

A total of 325 patients were enrolled in this study. The age range was 19–90 years, with mean of 54.8 ± 14.1 years; 132 cases (40.6%) were men and 193 cases (59.4%) women. There were: 1) 76 cases in the lithotomy position, including 42 cases of gynecological surgery and 34 cases of colorectal surgery, 2) 96 cases in the lateral position, including 47 cases of thoracic surgery and 49 cases of urology, 3) 102 cases in the prone position, all of which were spinal surgery, and 4) 51 cases in the supine position, including 24 cases of general surgery gastrointestinal surgery, 8 cases of breast surgery, 10 cases of cardiac surgery saphenous vein surgery, and 9 cases of lower limb arthroscopy. The general information of all patients in different positions was shown in Table 1.

**Table 1:** General information of all patients in the four surgical positions (*n* = 325)

Variable	Category	Position			
		**Lithotomy position (*n* = 76)**	**Lateral position (*n* = 96)**	**Prone position (*n* = 102)**	**Supine position** **(*n* = 51)**
Age(Mean ± SD)		43.8 ± 12.5	54.9 ± 12.6	62.8 ± 12.3	51.6 ± 15.4
Sex	Male	22(28.9%)	58(60.4%)	48(47.1%)	24(47.1%)
	Female	54(71.1%)	38(39.6%)	54(52.9%)	27(52.9%)
BMI		22.5 ± 3.3	24.3 ± 3.3	25.3 ± 3.6	23.9 ± 3.7
Preoperative sitting position eye pressure	Left	18.1 ± 2.2	17.6 ± 3.0	19.4 ± 3.2	18.0 ± 3.0
	Right	17.8 ± 2.0	17.3 ± 2.7	19.6 ± 2.1	18.5 ± 3.7
Previous medical history		24(31.6%)	17(17.7%)	71(69.6%)	17(33.3%)
Duration of operation(h)		2.5 ± 1.3	3.0 ± 1.3	3.5 ± 1.2	2.1 ± 0.9

Between four surgical positions, there was no statistical difference (*P* > 0.05) in the right and left IOP, heart rate, systolic blood pressure, diastolic blood pressure, and oxygen saturation after the patients were admitted to the room (Table 2).

**Table 2:** Comparison of blood pressure and vital signs of patients admitted to the room in four surgical positions (*n* = 325)

Variable	Category	Position				***P***
		**Lithotomy position (*n* = 76)**	**Lateral position (*n* = 96)**	**Prone position (*n* = 102)**	**Supine position** **(*n* = 51)**	
Eye pressure mean ± std (Range:min, max)	Left	18.5 ± 2.6 (11, 26.7)	19.3 ± 4.2 (10.2, 31)	19.9 ± 3.6 (12.1, 30.5)	19.1 ± 3.3 (12.6, 27.1)	0.55
	Right	18.2 ± 2.7 (9.5, 25.1)	18.9 ± 3.4 (10.6, 27.5)	20.0 ± 3.4 (11.6, 28.5)	18.9 ± 3.6 (11.1, 27.2)	0.26
Heart rate		75.3 ± 11.6	74.3 ± 12.3	72.9 ± 12.4	75.3 ± 11.4	0.54
Systolic blood pressure		123.0 ± 15.5	143.4 ± 22.8	148.3 ± 24.3	140.4 ± 21.6	0.09
Diastolic blood pressure		71.5 ± 9.4	80.5 ± 10.5	84.2 ± 70.6	78.9 ± 11.3	0.22
Oxygen saturation		99.3 ± 1.1	98.4 ± 1.8	97.9 ± 1.7	97.1 ± 12.5	0.11

### Patterns of IOP changed with surgery duration in 4 surgical positions

**Fig. 1:**
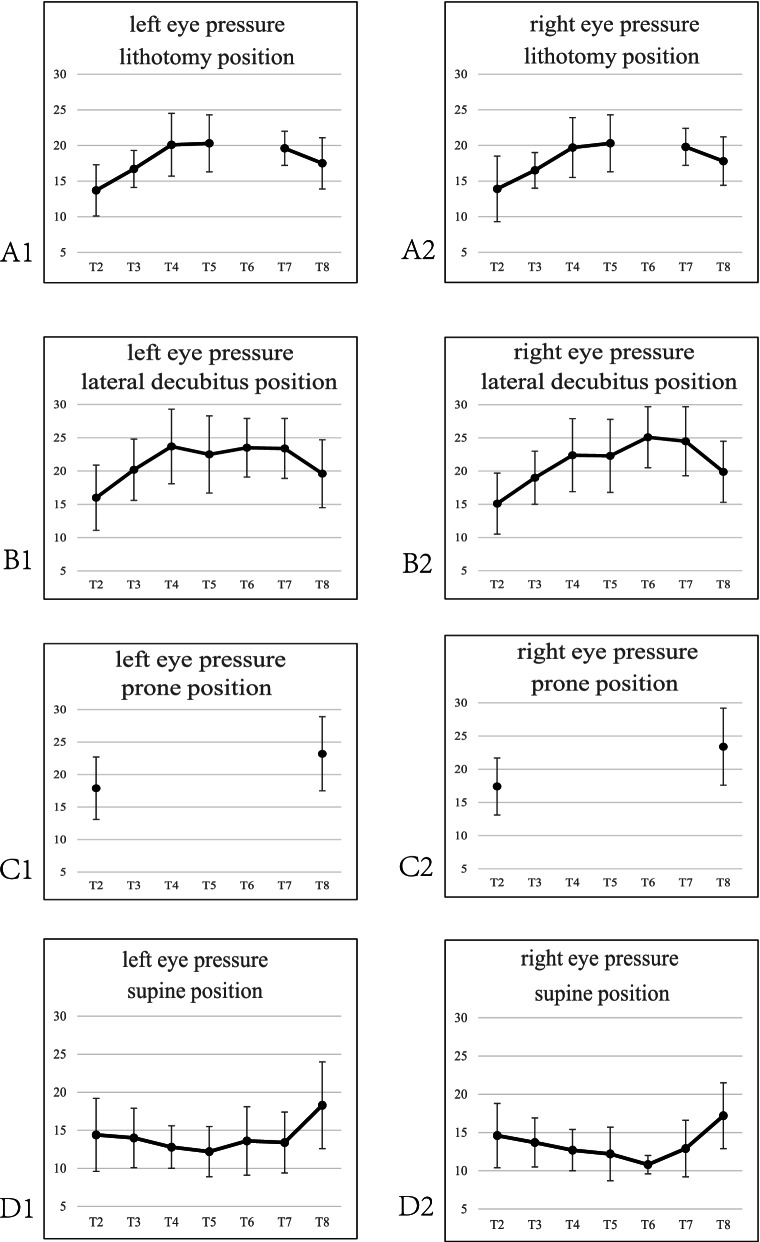
IOP of left and right eyes on different surgical positions with time. Note: **A1**: IOP of left eye on lithotomy position; **A2**: IOP of right eye on lithotomy position; **B1**: IOP of left eye on lateral decubitus position; **B2**: IOP of right eye on lateral decubitus position; **C1**: IOP of left eye on prone position; **C2**: IOP of right eye on prone position; **D1**: IOP of left eye on supine position; **D2**: IOP of right eye on supine position. T2: 10 min after general anesthesia in the supine position, T3: 10 min after changing position, T4: 1 h after the start of surgery, T5: 2 h after the start of surgery, T6: 4 h after the start of surgery, T7: 10 min before the end of surgery, T8: before leaving the operating room

The surgical position changed through three stages with the surgical process: flat (anesthesia)—position change (position required for surgery)—flat (awake and extubate at the end of surgery). IOP changes with different surgical positions were shown in Fig. 1. IOP of both left and right eyes respectively at each time point in the head-down lithotomy position, lateral position, and supine position showed statistical differences by ANOVA test (*p* < 0.05), and IOP of both left and the right eyes in the prone position before exit from the operating room were significantly higher than that 10-min after anesthesia (*p* < 0.01). From the beginning of the operation until the patients left the operating room, 51.5% of IOP exceeded 24 mmHg, along with 34.2% in the lithotomy position, 89.4% in the lateral position, and 48.5% in the prone position, and 13.7% in the supine position. With the operation progression, IOP rose to the highest 2 h after the start of lithotripsy, with 38.2% of IOP approaching or exceeding 24 mmHg; 4 h after the start of lateral surgery, 90.0% of IOP exceeding 24 mmHg on the compressed side; and before the patients with prone position left the operating room, 43.1% of IOP exceeding 24 mmHg.

### Effect of different angular on IOP in head-down and foot-up surgical position

During gynecological surgery, the head-down and foot-up surgical position was placed after the pneumoperitoneum was established. In this study, the angle of head down was divided into three groups: < 20° (34 patients), 20–30° (17 patients), and > 30° (24 patients). The mean intraocular pressure in both eyes of the three groups was 15.7 ± 2.3 mmHg, 16.9 ± 1.6 mmHg, and 17.5 ± 2.5 mmHg, respectively, 10 min after head-down and foot-up surgical position. The IOP under different postural angles showed statistical differences (*F* value = 4.85, *P* < 0.05), and the larger the head-down angle, the higher the IOP.

### IOP between compressed eye and non-compressed eye in the lateral position

IOP on the compressed side in the lateral position was higher than that on the non-compressed side after the position change until the exit from the operation room, and the difference was statistically significant (*p* < 0.01) (Table 3).

**Table 3:** IOP difference between compressed eye and non-compressed eye in the lateral position at each time point

Time point	N	Compressed eye (mean ± SD)	Non-compressed eye (mean ± SD)	IOP difference (mean ± SD)	t	*P*
T0	96	17.32 ± 2.74	17.33 ± 2.86	0.31 ± 2.26	1.34	0.18
T1	96	19.13 ± 3.88	19.11 ± 3.73	0.02 ± 3.44	0.06	0.96
T2	96	15.59 ± 4.22	15.52 ± 5.31	0.08 ± 3.62	0.21	0.83
T3	96	21.13 ± 4.49	18.11 ± 3.54	3.02 ± 4.01	7.38	< 0.01
T4	96	24.68 ± 5.50	21.48 ± 5.14	3.21 ± 4.31	7.29	< 0.01
T5	92	23.93 ± 6.17	20.84 ± 4.53	3.09 ± 5.05	5.87	< 0.01
T6	42	26.23 ± 4.65	22.40 ± 3.61	3.83 ± 4.79	5.18	< 0.01
T7	94	24.87 ± 5.17	23.04 ± 4.47	1.71 ± 4.27	3.89	< 0.01
T8	96	19.65 ± 5.06	19.88 ± 4.64	1.16 ± 3.63	3.12	< 0.01

IOP difference between the eyes = IOP in the compressed side—IOP in the non-compressed side

### Univariate and multivariate analysis of intraocular pressure variation

The univariate analysis revealed that prone position, lateral position, Lithotomy position, baseline IOP, duration of operation, infusion amount during surgery, and access volume was correlated with IOP changes. In the multivariate linear regression analysis adjusted by other factors, postural position and baseline IOP were associated with IOP differences between before and after surgery (Table 4).

**Table 4:** Univariate and multivariate analysis of the IOP difference between after and before surgery

Variable	Univariate		Multivariate	
	**β**	***P***	**β**	***P***
**Position**				
Supine position	Ref		Ref	-
Prone position	7.019	< 0.01	9.300	< 0.01
Lateral position	10.399	< 0.01	11.230	< 0.01
Lithotomy position	4.187	< 0.01	3.626	< 0.01
Heart rate after admission	0.022	0.44		
Systolic pressure after admission	0.027	0.07		
Diastolic pressure after admission	0.012	0.16		
Oxygen saturation after admission	-0.006	0.93		
Baseline intraocular pressure	-0.543	< 0.01	-0.778	< 0.01
Sex				
Male	1.234	0.08		
Previous medical history				
Yes	-0.531	0.46		
BMI	0.176	0.07		
Duration of operation(h)	0.771	< 0.01		
Use of hormone drugs	-1.749	0.47		
Age	0.034	0.15		
Infusion volume during surgery	0.002	< 0.01		
Urine volume during surgery	-0.001	0.15		
Blood loss during surgery	0.002	0.12		
Access volume	0.002	< 0.01		

Baseline IOP was the IOP at 10 min of lying down after general anesthesia. The IOP difference in different positions before and after surgery was calculated as follows: 1) prone position: the IOP difference between before exiting the operation room and 10 min of lying down after general anesthesia; 2) all of supine position, lateral position, and Lithotomy position: the IOP difference between the end of surgery and 10 min of lying down after general anesthesia. IOP in lateral position was the IOP on the compressed side, otherwise, it was the mean on both sides in other positions. BMI = weight/height^2^ (kilograms /m^2^)

## Discussion

In this study, IOP was measured in four surgical positions during the operation, which showed different trends with the surgical process and position changes. Postural position for the surgery played a role in IOP change. After postural changes, the body maintained hemodynamic stability through a series of complex regulatory mechanisms, including the self-regulation system, venous and arterial systems, and neural reflexes. However, this regulatory mechanism was weakened under anesthesia, so healthcare workers needed to understand the pattern of IOP changes in different surgical positions.

Among the four position groups, it was found that the patients in the prone position were older and had the highest preoperative BMI and the highest IOP in the preoperative sitting position. However, there was no significant difference in both IOP and vital signs among the four surgical positions after admission, which might be related to the fact that the positions were adjusted to supine position and the patients were generally tense, resulting in increased blood pressure. Previous studies had shown that IOP increased by 0.3–6.0 mmHg in the general population when they changed from a sitting position to a supine position [13]. In our study, the average change of IOP increase in the four groups was 0.4–1.7 mmHg after changing from the preoperative (sitting position to the postoperative supine position), which was within the range reported in the literature. IOP after anesthesia was lower than before anesthesia in all four groups, which was related to the anesthetic medication [14]. Anesthesia induction with midazolam, propofol, fentanyl, and cis-atracurium had direct or indirect effects on IOP lowering [15].

In this study, all the surgeries on the lithotomy position were pelvic or abdominal surgeries using laparoscopic techniques. The surgical position after the CO_2_ pneumoperitoneum was changed to have different angles with head-low and foot-high positions. After 10 min of position change, IOP tended to rise, which was consistent with the findings of Shuangquan Zhang and Yueqin Miao [16, 17]. The reasons were that: 1) the thoracic pressure increased due to diaphragmatic elevation after the CO_2_ pneumoperitoneum; 2) the anesthesiologist increased the ventilation volume/min to maintain PetCO_2_ in a reasonable range and the airway pressure elevation was transmitted to the thoracic cavity, increasing central venous pressure (CVP) [16]; 3) the low head position also increased the volume of blood returning to the heart, which increased the venous pressure in the head and face, causing obstruction of venous return in the eye and head and resulting in the pressure increase in episcleral vein, leading to pressure increase in IOP [12, 18].

IOP after position change tended to increase slightly with the prolongation of surgery time Fig. 1 (A1, A2). IOP in both the left and right eyes reached the highest 2 h later and 38.2% of IOP exceeded 24 mmHg. IOP decrease at the end of surgery was associated with the recovery of position and the end of pneumoperitoneum. By analyzing IOP means in different angles of head-down position, it was found that IOP mean was 15.7 mmHg, 16.9 mmHg, and 17.5 mmHg at head-down < 20°, 20–30°, and > 30°, respectively, at 10 min after a position change. Nevertheless, 17.8 mmHg was measured at 40° head-down at 30 min after pneumoperitoneum in previous studies, which showed an IOP increase with increasing head-down angle. Therefore, the increase in head-down angle could be associated with a mild change in the intraocular pressure. While the short-term angle increase could not result in the extreme elevation of IOP.

Both left and right IOPs increased at 10 min after changing to the lateral position, which was consistent with Malihi finding that lateral position caused IOP to increase [19]. Hwang studied the IOP change in lateral position for pulmonary surgery under general anesthesia and showed that IOP mean in lateral position was 2 mmHg higher than that in the supine position, and the difference increased to 4 mmHg after 30 min and remained stable after 150 min [20]. Figure 1 (B1, B2) showed that IOP was significantly higher at 10 min after a position change, 1 h after the start of surgery, 2 h, 4 h, and before the end of surgery than IOP at 10 min after anesthesia in the supine position. The IOP mean in the left eye increased by 4.2 mmHg at 10 min after a position change, which remained stable to the end. IOP in the right eye had the same trend as in the left eye. And IOP in both eyes in the lateral position was close to or higher than pathological IOP (24 mmHg) 4 h after surgery and before the end of surgery. Because all surgeries were performed in the lumbar bridge lateral position with the head below the midline of the torso, which blocked the venous return to the head and face, causing pressure increase in the episcleral vein and thus an increase in intraocular pressure. And it also was related to the redistribution of blood volume around the eyes, such as the simultaneous rise in choroidal blood volume and orbital venous pressure. In addition, in table 3, there was no significant difference in IOP between the two eyes before the position change, while after changing to the lateral position, IOP on the compressed side was significantly higher than the uncompressed IOP value. It suggested an association with increased choroidal blood flow and obstructed reflux on the compressed side, which is consistent with the findings of Wang Wei [21]. By 4 h of surgery (T6), the IOP mean on the compressed side was 26.7 ± 3.6 mmHg, and the difference in IOP between the two eyes reached approximately 4 mmHg, with 90.0% of IOP on the compressed side exceeding 24 mmHg. It meant the compressed eye has a possibility to have an extreme elevation of IOP. Therefore, the nurses in the operation room should pay attention to the placement of the lateral recumbent lumbar bridge position. Direction compression should be avoided. And it’s encouraged to reduce the head-low angle or keep the upper body supine under the premise of ensuring the operation of the physician.

Intraoperative prone IOP values were missing because the rebound IOP meter could not measure the IOP of patients in the prone position. But Fig. 1 (C1, C2) showed that IOP after the patients were turned to the supine position at the end of the surgery, was still higher than that before anesthesia; the difference was statistically significant. The IOP mean in both eyes before the exit was 23.1 ± 5.7 mmHg, and 43.1% of patients had an average IOP of more than 24 mmHg, indicating that the prone position still significantly elevated IOP. Lee retrospectively analyzed 93 patients with concomitant blindness after prone spine surgery from the American society of anesthesiologists (ASA) registry and found that their average anesthesia time was 9.8 ± 3.1 h, with 94% of patients having greater than 6 h of surgical anesthesia [22]. The longer the duration of surgery, the more attention should be drawn to ocular protection by health care providers. The causes of elevated intraocular pressure in prone position were related to the intraoperative use of a horseshoe-shaped headrest, which directly acted on the eye to increase intraocular pressure [23]. This was also the most common cause of central retinal artery obstruction. The change in the head's center of gravity due to the prone position, which had a large amount of fluid in the optic nerve tissue space, led to increasing pressure around the eyes and further compresses the veins to increase intraocular pressure [24]. In addition, in the prone position, the weight of the human trunk was mainly borne by the cephalad, anterior thorax, and abdomen, which did not have the advantage of weight-bearing anatomically. The position change, which resulted in the redistribution of blood, the increase in choroidal blood perfusion, and the increase in ocular perfusion pressure, finally led to an increase in IOP [25].

The majority of the POVL cases that had been reported were operated in the prone position. In this study, IOP in the prone position was significantly higher compared with other positions before the patient exited the room, suggesting that the prone position may have a greater effect on IOP than other positions.

Only 15.7% of the patients in this study in the supine position underwent a short adjustment of head high, head low, left high, or right high after the start of surgery, and the rest did not undergo postural adjustment. IOP began to decrease after anesthesia and tended to decrease smoothly intraoperatively as the procedure progressed, with significant differences in IOP changes before and after surgery (Fig. 1, D1, D2). This trend was different from IOP change in the non-anesthetized state with the same position in the general population. IOP in the general population does not increase or decrease with time. Therefore, this change should be associated with the continuous use of anesthetic drugs intraoperatively [26]. Before exiting the room, the patient was awake and had elevated IOP, which reached the pre-anesthesia IOP level.

After multifactorial analysis, the surgical position was one of the risk factors affecting IOP elevation, and the lateral recumbent position had the greatest effect on IOP. It was possibly related to simply taking the pressure side IOP for analysis. The prone position and the lithotomy position also had a great effect on IOP, but due to the limitations of the measurement tools in this study, the values of IOP before the end of surgery were not collected in the prone position, thus possibly interfering with the determination of the effective impact of the prone position on IOP. This study also found that the baseline IOP after anesthesia was negatively correlated with IOP elevation before and after surgery, suggesting paying attention to the effect of medications on IOP fluctuations during induction of anesthesia and to lower pressure as smoothly as possible.

The ASA POVL Task Force concluded that there was insufficient evidence that heavy fluid infusion was associated with blindness after spine surgery, but suggested that crystalloid fluid should be infused in an appropriate ratio with colloid fluid to maintain blood volume [27]. However, the amount of fluid infusion was associated with IOP elevation in the univariate analysis, while this variable was excluded in the multifactorial analysis. It was consistent with the conclusion of the ASA POVL Task Force. Nevertheless, the influence of patients' factors such as gender, age, and previous medical history (hypertension, diabetes, etc.) on post-spinal surgery complications of ION was controversial [28–30]. And gender, age, BMI, and previous medical history (well-controlled hypertension, diabetes, appendicitis, etc.) were not influential factors for IOP changes in this study.

There were some limitations in our study. Due to measurement limitations of the IOP meter, intraoperative IOP changes in the prone position could not be measured. Although no patients complained of postoperative ocular discomfort in this study, the measurement and follow-up of ocular adverse effects were lacking. In the next study, ocular discomfort should be followed up postoperatively.

## Conclusion

Different surgical positions had certain effects on the IOP of patients, showing different patterns of changes with the surgical process. Therefore, if the intraoperative head-low position was involved, the head-low angle should be minimized without affecting the surgical operation. Direct pressure should be avoided on the eyeball in prone position; the head frame was suggested to be used to fix the head to facilitate the venous blood return to the head and reduce the magnitude of intraoperative IOP changes. Intraoperative IOP measurement was recommended for patients in lateral recumbent, head-low foot-high lithotomy position, and prone position. After the operation, the nurses should make a good handover and pay attention to the postoperative IOP changes and the occurrence of any adverse events, to identify the problems early and provide appropriate treatment.

## Data Availability

The datasets generated and/or analysed during the current study are available in the Baidu Netdisk repository, Weblink: https://pan.baidu.com/s/1m4_ITnGs-sLKRRc6qtBLaQ; Code: hj8i.
